# Late antibody-mediated rejection in a kidney transplant recipient: COVID 19 induced?

**DOI:** 10.1186/s12882-022-02713-x

**Published:** 2022-03-05

**Authors:** Nicole Nourié, Hussein Nassereddine, Sarah Mouawad, Louaa Chebbou, Rita Ghaleb, Fatmeh Abbas, Hiba Azar

**Affiliations:** 1 Nephrology Department, Saint Joseph University, Hôtel-Dieu de France, Beirut, Lebanon; 2 Pathology Department, Saint Joseph University, Hôtel-Dieu de France, Beirut, Lebanon; 3 Department of Pathology and Laboratory Medicine, American University of Beirut Medical Center, Beirut, Lebanon

**Keywords:** COVID 19, Antibody mediated rejection, Natural killers, Case report

## Abstract

**Background:**

Antibody-mediated rejection (AMR) was described in kidney transplant patients after viral infections, such as the cytomegalovirus. Very few cases were recently reported after severe acute respiratory syndrome coronavirus 2 (SARS-CoV-2) infection, probably in the context of lowering of immunosuppressive therapy. To date, no direct immunological link was proved to explain a connection between the coronavirus disease 19 (COVID-19) infection and antibody-mediated rejection (AMR) if it exists.

**Case presentation:**

Here we try to find this association by presenting the case of a low immunological risk patient who presented, six years post-transplant, with c4d negative antibody mediated rejection due to an anti-HLA-C17 de novo donor specific antibody (DSA) after contracting the coronavirus disease 19.

The HLA-Cw17 activated the antibody-dependent cell-mediated cytotoxicity via the KIR2DS1 positive NK cells.

**Discussion and conclusions:**

This case report may prove a direct role for COVID-19 infection in AMRs in the kidney transplant recipients, leading us to closely monitor kidney transplant recipients, especially if they have “at-risk” donor antigens.

## Background

Since the early onset of the COVID-19 pandemic, special considerations were attributed to kidney transplant recipients, who are at an increased risk of contracting the virus, developing a severe disease course and worsening of their kidney function. On the other hand, acute kidney injury in patients hospitalized for a COVID-19 infection is very frequently encountered with a prevalence reaching up to 90% in patients on mechanical ventilation, leading to an increased morbidity and mortality [1]. Mechanistically, acute kidney injury is most likely due to acute tubular necrosis but cases of collapsing glomerulopathy, podocytopathies, anti-neutrophil cytoplasmic antibody vasculitis, and anti-glomerular basement membrane disease has been reported. Moreover, endothelial cell injury manifested as hypertension, prothrombotic injuries, and thrombotic microangiopathy has also been described [2]. However, little is known of the direct immunological consequences of this virus on transplanted kidneys. Here we present the case of an antibody-mediated kidney rejection soon after COVID 19 infection in a kidney transplant recipient and try to link it to the virus’s direct involvement.

## Case presentation

### Case review

This is the case of a 54-year-old male patient who received a kidney transplant from a living donor, his wife, in December 2014. His underlying nephropathy was a focal and segmental glomerulosclerosis, presumed secondary to a genetic mutation since he had a strong family history of kidney disease, even though no genetic testing was performed. He had been on hemodialysis for 6 months before transplantation. He had no previous blood transfusions, his panel reactive antibodies (PRAs) was negative and he had 5 mismatches in class I and was fully matched in class II. He received an induction therapy with Basiliximab and was maintained on tacrolimus, mycophenolate mofetil (MMF), and low- dose steroids. His immediate post-operative period was uneventful with a serum creatinine of 1.1 mg/dl upon discharge. He continued his regular out-patient follow-up, with no major events, a stable kidney function, and no proteinuria up until August 2020. In September 2020, he presented with fatigue, fever and tested positive with COVID-19 infection. His respiratory symptoms were mild with no oxygen requirements; therefore, he was treated with acetaminophen and oral hydration. His low-grade fever persisted for more than one week, so a chest CT was ordered and showed multiple well-defined ground-glass opacities in both lungs consistent with COVID-19 infection. A blood test was done then and showed a c-reactive protein of 14 mg/l, white blood cells of 5.3 × 10^9^/L with 70% neutrophils and 20% lymphocytes, and a serum creatinine level of 1.4 mg/dl. Since the patient presented with a mild case of COVID-19 he was not treated with dexamethasone, monoclonal antibodies or any other medications. His MMF was decreased from 1500 to 1000 mg per day for a total of 10 days, and his tacrolimus trough levels were kept within target range (6.8 ng/ml). His serum creatinine returned to its baseline of 1.2 mg/dl after resolution of the infection. Later on, it started to fluctuate on higher values reaching 1.6 mg/dl in April 2021, with no evident explanation. There was no introduction of new medications, no intercurrent infections, and again a stable tacrolimus trough level. He was therefore admitted for a kidney biopsy.

### Biopsy and testing results

**Fig. 1:**
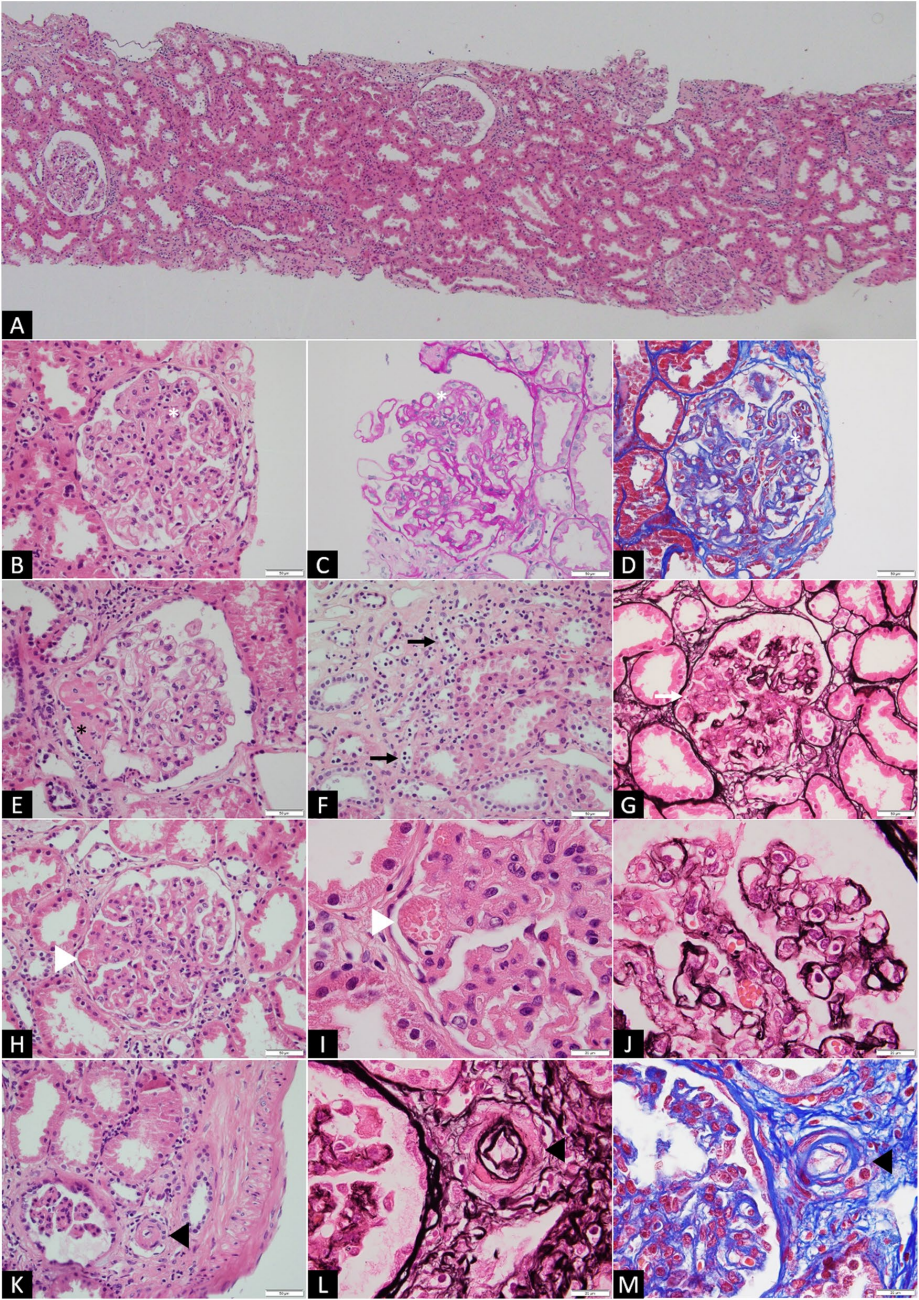
Biopsy findings. Light microscopy showing (**A**) no significant interstitial inflammation (i0) (hematoxylin and eosin – HE, × *4* magnification). Higher magnification displaying (**B**, **C**, **D**) glomerulitis (white asterisk, HE, PAS, Trichrome, × *40* magnification), (**E**) peri-hilar focal segmental glomerulosclerosis (black asterisk, HE, × *40* magnification), (**F**) moderate peritubular capillaritis (black arrows, HE, × *40* magnification), (**G**) segmental mesangiolysis (white arrow, Jones, × *40* magnification), (**H**, **I**, **J**) a thrombotic microangiopathy (TMA) affecting a glomerulus with fragmented red blood cells (white arrowhead, HE, × *40* and × *100* magnification) and glomerular basement membrane remodeling with double contours and mild endothelial swelling (Jones, × *100* magnification), (**K**, **L**, **M**) TMA affecting a small arteriole with endothelial swelling in the lumen (black arrowhead, HE, Jones, Trichrome, × *40* and × *100* magnification)

**Fig. 2:**
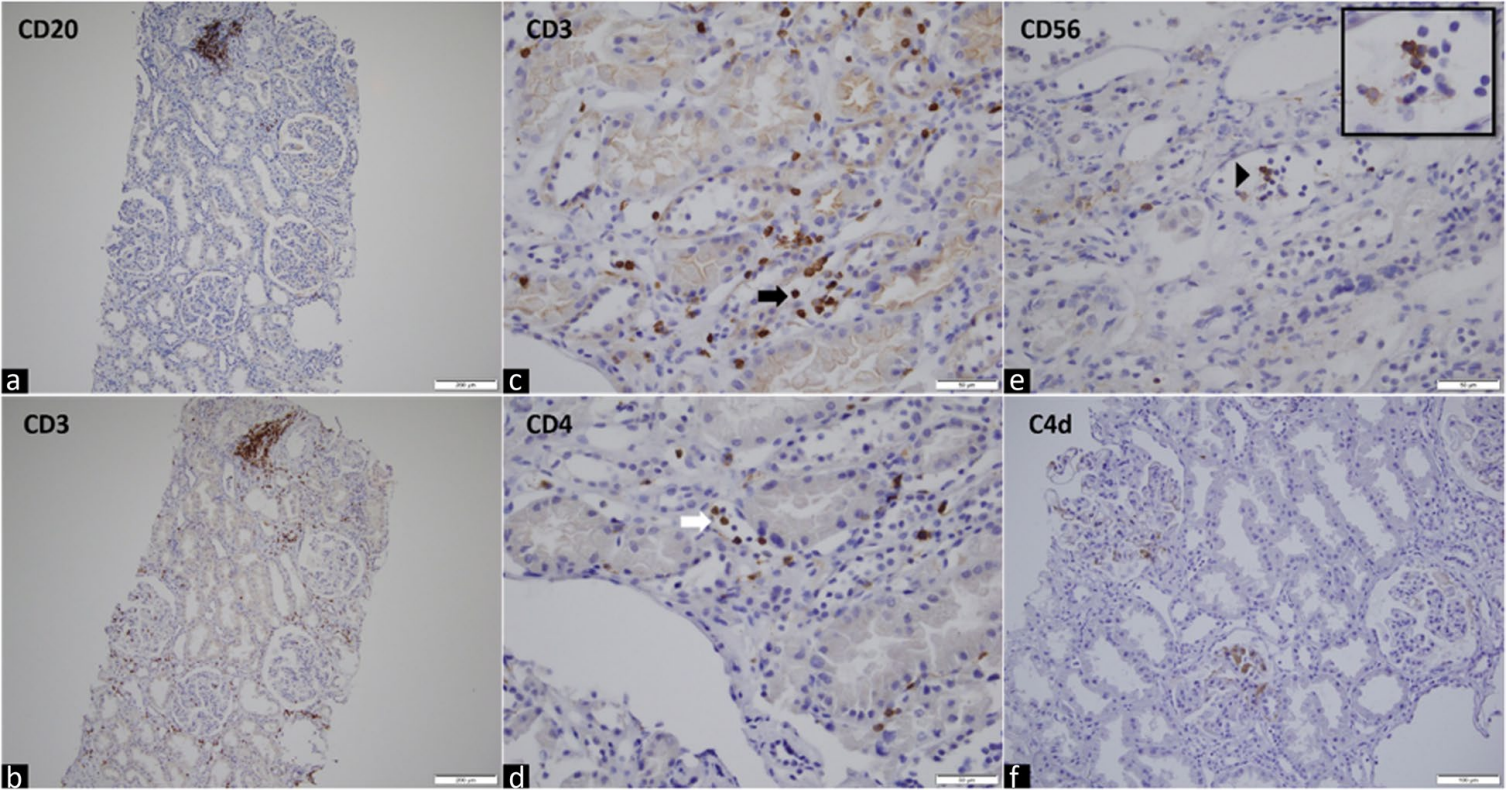
Immunohistochemical analysis. (**a**,**b**) Immunostaining for CD20 and CD3 showing only focal interstitial inflammation. (**c**,**d**,**e**) Immunostaining for CD3, CD4, and CD56 showing capillary to contain T cells (CD3 + , CD4 +). Some cells expressed CD56 (CD56 +). (**f**) There was no C4d staining of peritubular capillaries (C4d0)

Light microscopy showed histological evidence of global glomerulitis (g2), moderate capillaritis (ptc2) and thrombotic microangiopathy (TMA) affecting arterioles and glomeruli (Fig. 1). A single focus of tubulitis was noted (t0). These histopathological findings were consistent with an active AMR. Interestingly, there was no C4d staining of peritubular capillaries or vasa recta upon immunohistochemistry (C4d0). Only focal interstitial inflammation (i0, < 10% of unscarred cortical parenchyma) was noted with CD3 and CD20 immunostaining. Capillaries contained mainly CD3^+^ and CD4^+^ T-cells, with some CD56^+^ cells (Fig. 2). Direct immunofluorescence studies showed IgM, C3, and C1q deposition in a segmental distribution in the lesioned glomeruli, consistent with focal and segmental glomerulosclerosis. Some podocytes protein resorption droplets were highlighted by IgA, Kappa, and Lambda. There was no significant IgG deposition nor mesangial IgA deposits. Donor-specific antibodies came back positive for class I, HLA-Cw17, with an MFI of 6689 confirming the diagnosis of late active c4d negative AMR.

The patient was also tested for killer cell Ig-like receptors (KIRs) genotyping using the KIR SSO Genotyping Test (One Lambda) which applies Luminex® technology, and came back positive for the expression of KIR2DS1.

### Treatment and follow-up

The patient was treated with pulse steroids, five sessions of plasma exchange and IVIG with a total dose of 2 g/kg, with a good response to treatment and a creatinine at 1.3 mg/dl upon discharge. His DSA decreased three months later to an MFI of 2710, with a delta MFI of 60%.

## Discussion and conclusions

The kidney transplant population displays a high risk of mortality, when infected with COVID-19, with numbers reaching 26 to 28% in various reports across the United States and Europe compared to the 1 to 5% mortality in the general population [3, 4]. These negative outcomes lead many nephrologists to adjust the baseline immunosuppression regimen when their patients get infected. The most common approach is reducing or withholding anti-proliferative agents or mammalian target of rapamycin inhibitors, and/or aiming for lower trough levels of calcineurin inhibitors [5]. This reduction in immunosuppression proved to be safe, with no rejection episodes, no de novo DSAs, or significant changes in panel reactive antibodies (PRAs) [6].

Antibody-mediated rejection (AMR) remains a main cause of allograft loss, triggered by the presence of antibodies directed against donor antigens. These antibodies can preexist prior to transplantation, in the context of previous transplantation, pregnancy or blood transfusions, or appear later on. De novo antibodies (DSAs) can appear secondary to rapid or excessive minimization of immunosuppression, non-adherence to immunosuppressive medication, or after infection via molecular mimicry.

Our patient presented with an anti-HLA-C class I DSA, which is very unusual in late AMR. In fact, de novo DSAs are, in up to 75% of cases, class II antibodies, especially DQ type. Class I antibodies are detected sooner after transplant and are usually complement binding, IgG1 or IgG3 with C4d deposits which is not the case in our patient [7]. Non-complement binding DSA can cause damage via activation of the innate immune cells such as Natural killer cells that bind to Fc fragments of DSA causing endothelial cell injury. DSA can also increase the production of the vascular endothelial growth factor causing endothelial proliferation [8].

Our patient presented with histological evidence of AMR, along with FSGS lesions probably hyperfiltration-mediated in a long-standing hypertensive kidney graft recipient. The TMA lesion can occur in the setting of acute humoral rejection, and viral infections, particularly SARS COV2 which was described to be responsible of direct endothelial injuries.

Interestingly, in our case, capillaries were found to contain some CD56^+^ cells. CD56 is present over most natural killer (NK) cells and a limited subpopulation of T cells.

On the other hand, the HLA system plays an important role in the outcome and severity of many infectious diseases such as the human immunodeficiency virus [9] and the malaria parasite [10]. This role is pronounced in class I molecules that are expressed on all nucleated cells, and play a central role in immune regulation by presenting cytoplasmic peptides and in particular viral antigens. HLA-C locus, in particular, is expressed at lower levels on the surface of nucleated cells in comparison to HLA-A and HLA-B loci and is less polymorphic [11]. This sub-class is a major determinant of natural killer (NK) cell activity secondary to its pronounced interactions with killer cell Ig-like receptors (KIRs) [12]. The polymorphism of the receptor (KIR) and its ligand (HLA class I) dictate the consequence of such interaction. In humans, KIR receptors have four types of epitopes, two of them present on HLA-C loci. The C1 epitope present on HLA-C allotypes with Asparginine at position 80 of the α1 domain, and C2 epitope present on HLA-C allotypes with Lysine at position 80 [13], such as HLA-Cw17. The C2 epitope is recognized by both KIR2DS1 and KIR2DL1. KIR2DS1 is short for killer cell immunoglobulin-like receptor with two Ig domains and a short cytoplasmic tail, and is a subgroup of KIRs that has an activating function, while KIR2DL1 has a long cytoplasmic tail with an inhibitory function. With a hypothesis of viral-induced upregulation of HLA-C17 and its’ interaction with KIR2DS1 on NK cells, we performed KIR genotyping for the expression of KIR2DS1 and it returned positive.

In kidney transplantation, antibody-dependent cell-mediated cytotoxicity (ADCC) mediated by NK cells is associated with humoral graft vasculopathy [14].

Very interestingly, in recently published data, HLA-Cw17 was found to be associated with severe outcome and higher intensive care unit admission rate in patients with COVID-19 infection [15, 16] with mainly a higher risk of cardiovascular complications pointing out to endothelial dysfunction. On another note, HLA-Cw17 is a very infrequent allele, with an even lower prevalence in our population (*f* = 0.0011) [17].

Our patient who is considered of low immunological risk with no pre-transplant DSA, was very compliant to his medications and showed regularly on his follow-up visits. Yet, he presented with an AMR six years after his kidney transplantation. His kidney function started to worsen after he contracted a COVID-19 infection, his illness course was considered benign, with no oxygen needs and no hospital admission. The kidney biopsy showed signs of microvascular inflammation and TMA with no c4d staining, and his DSA came back positive with antibodies directed towards HLA-Cw17. This antigen was proved to correlate with worse outcomes in patients infected with COVID-19, especially on a cardiovascular level, bringing us full circle back to endothelial injury.

Thus, the HLA-Cw17 via its C2 epitope could have presented the cytoplasmic COVID-19 viral antigen to the activating KIR2DS1 positive NK cells in our patient, thus activating the antibody-dependent cell-mediated cytotoxicity. This reasoning may prove a direct role for COVID-19 infection in AMRs in the kidney transplant recipients, leading us to closely monitor kidney transplant recipients, especially if they have “at-risk” donor antigens.

## Data Availability

All data and material presented in the article are present in our medical files and are available from the corresponding author on reasonable request.

## Abbreviations

ADCC: Antibody-dependent cell-mediated cytotoxicity
AMR: Antibody-mediated rejection
COVID-19: Coronavirus disease 19
DSAs: De novo antibodies
KIR: Killer cell Ig-like receptor
MMF: Mycophenolate mofetil
NK: Natural killer
PRA: Panel reactive antibodies
SARS CoV-2: Severe acute respiratory syndrome coronavirus 2
TMA: Thrombotic microangiopathy
